# Monetary Expansion and the Banking Lending Channel

**DOI:** 10.1371/journal.pone.0164338

**Published:** 2016-10-07

**Authors:** Benjamin Miranda Tabak, Tito Belchior Silva Moreira, Dimas Mateus Fazio, André Luiz Cordeiro Cavalcanti, George Henrrique de Moura Cunha

**Affiliations:** 1 Department of Law and Economics, Catholic University of Brasília, Brasília, Distrito Federal, Brazil; 2 Department of Economics, Catholic University of Brasília, Brasília, Distrito Federal, Brazil; 3 London Business School, London, UK; 4 Department of Business, Catholic University of Brasília, Brasília, Distrito Federal, Brazil; 5 Department of Economics, Catholic University of Brasília, Brasília, Distrito Federal, Brazil; East China University of Science and Technology, CHINA

## Abstract

This paper examines the bank lending channel, which considers how monetary authority actions affect the variation of loans. We focus on the BRICS (Brazil, Russia, India, China and South Africa) totalizing 1254 banks from five countries in the period 2000–2012 (totalizing 13 years). The empirical results show that the effect of money supply growth on the growth of loans is non-linear and inverted U-shaped. In this context, our results show empirical evidence expansionary monetary policies do not increase the propensity of economic agents to systematically take greater risks on the market. After a certain level of money stock, increases in the money supply do not lead to increased negotiated credit.

## Introduction

The recent financial crisis has reinforced the interest of authorities and researches about how monetary policy affects banks’ behavior more specifically credit [1] and risk [2]. This issue becomes more important when central banks are making use of unconventional monetary policies—other than directly changing the short-term rate of the economy—to affect the real economy. Most of the papers on this topic so far have analyzed how banks respond to changes in policy rates. In addition, most of the attention has been focused on developed economies. We are going to contribute to the literature by studying how banks from the current leading emerging economies, i.e. BRICS (Brazil-Russia-India-China-South Africa), react to changes in the money aggregates in each country.

Using a dataset with 1254 banks from the 5 BRICS countries in the 2000–2012 period, this paper shows that the effect of an increase in the real balances of a country, measured by the ratio of the M1 or M2 monetary aggregates and the nominal GDP, has a non-linear effect on total lending. While banks increase credit as a response to moderate levels of increase in money supply, both conservative and expansionary monetary policies appear to harm them. A stark decrease in money supply may imply larger funding costs and thus a reduction in the loan portfolio. A very expansionary policy, on the other hand, might generate uncertainty and other macroeconomic shocks take make banks delay the issue of new loans.

As already highlighted the subprime crisis, which started in 2007, prompted even further the debate about the role of banks in the monetary policy transmission process. The bank credit has received much attention from researchers as a channel for monetary policy transmission, considering that monetary policy directly affects bank deposits, since deposits represent the supply of loan funds, which act as a driving force for credit.

In the above context, a restrictive monetary policy reduces the amount of deposits in the banking system leading to a decline in loans if banks realize that the payments of credits already granted will not be sufficient to restore the reduction of deposits based on a possible increase in defaults. This requires the banks to increase interest rates on new loans due to a decline in the supply of credit [3]

There are two traditional channels can explain the association between monetary policy and the evolution of the balances of bank deposits [4]. Central banks can change the level of deposits through the control of bank reserves and via manipulation of the money multiplier. [4] also advocates another approach in which the bank lending channel is made possible through the impact of monetary policy on the soundness of bank balance sheets and on the perception of risk.

The term "risk-taking channel of monetary policy was used to characterize the impact of monetary policy on market participants' desire to take on exposure to risk, in order to influence financial conditions and, ultimately, influence the economic decisions [2]. The authors show that changes in the financial system and in the prudent regulation can increase the importance of the risk-taking channel.

There has been a change from the traditional interest rate targeting approach to monetary policy, as observed in many central banks around the world and the Federal Reserve [5]. The main focus of the central banks is now their balance sheet. They have opted for the use of quantitative measures of monetary policy such as quantitative easing and credit easing.

[6] highlight the role of the banking sector to assess the impact of American monetary policy beyond its borders. The authors argue that adjustments in bank leverage act as a linchpin in the monetary transmission mechanism that works through fluctuations in risk-taking. In the international context, they find evidence of monetary policy spillovers on cross-border bank capital flows and the US dollar exchange rate through the banking sector.

The literature on monetary policy transmission mechanisms by means of bank loans has many approaches. It shows that an expansionary monetary policy, via reduction of the Central Bank’s basic interest rate, raises bank deposits leading to an increase in credit supply according to [3]. An alternative approach and more recent literature assumes that as the prime rate decreases, as a result of an expansionary monetary policy, the propensity of economic agents to take more risk on the market increases in line with the literature for risk-taking channel.

In general, this literature evaluates the transmission mechanisms of monetary policy through bank credit channels in a situation where the expansionary monetary policy, via reducing the basic interest rate, becomes able to increase the supply of credit. In this case, the Central Banks use the conventional monetary policy buying or sellinggovernment bonds. When the bank seeks to promote economic growth, itbuysgovernment bonds, which lowers short-term interest rates and increases the money supply.

When short-term interest rates are at or approaching zero, the expansionary monetary policy does not produce more effect. In this context, an endogenous increase in the money supply does not lead to a reduction of the interest rate that becomes sufficiently attractive to induce banks to increase the supply of credit. This would be a case known as "liquidity trap".

In this context, this strategy loses effectiveness and forces banks to try other strategies in order to stimulate the economy. However, the central bank can use unconventional instruments to improve the liquidity or the money supply in economy including forms of quantitative easing (QE). [7] argue that QE policies are those that unusually increase the monetary base, including asset purchases and lending programs. Programs designed to improve credit conditions—that is, credit easing—are a special case of QE if they also increase the monetary base.

One issue with this type of policy is that if the money supply increases too quickly, QE can lead to higher inflation rate. Furthermore, banks may decide to keep funds generated by quantitative easing in reserve rather than lending those funds to economic agents. Considering these arguments, we test the hypothesis that the effect of money supply growth on the growth of loans is non-linear and inverted U-shaped. In this context, there is a point of maximum that would be an inflexion point, which an increment of money supply will increase or keep funds in reserves rather than increase credit operations.

The literature has already conducted substantial research on recent quantitative easing programs. [8], [9], [10], [11] and [12], for example, study the Fed’s 2008–09 QE programs. [11] and [12] announcement study finds that large-scale asset purchase announcements reduced U.S. long-term yields. [13] indirectly calculate the effects of the Fed’s 2008–09 QE programs with a term structure model. Neely (2012) [14] evaluates the effect of the Fed’s 2008–09 QE on international long bond yields and exchange rates, showing that the effects are consistent with a simple portfolio balance model and long-run purchasing power parity. [15] constructed a model of money, credit and banking in which purchases of long-maturity government debt by the central bank are always a good idea, but for unconventional reasons. A floor system is preferred to a channel system, as a floor system permits welfare-improving asset purchases by the central bank.

[16] show that unconventional monetary policy in the United States appears to influence capital inflows to Brazil and, through this channel, its overall economic outlook and financial stability. Specifically, quantitative easing leads to capital inflows, exchange rate appreciation, stock market price increases, credit growth and expansion of domestic activity related to consumption. [17] examine gross financial inflows to developing countries between 2000 and 2013, with a focus on the potential effects of quantitative easing (QE) policies in the United States and other high-income countries. The authors find evidence for potential transmission of QE along observable liquidity, portfolio balancing, and confidence channels.

Despite this profusion of research on asset purchase programs and QE more generally in developed countries, there has been little attempt to analyze unconventional instruments of monetary policy and QE programs across central banks of the emerging economies, especially the BRICS. In this context, this article examines the bank-lending channel, which considers how monetary authority actions affect the variation of loans. We focus on the BRICS (Brazil, Russia, India, China and South Africa) totalizing 1059 banks from 5 countries in the period 2000–2012 (totalizing 13 years). The central bank influences the variation of loan supplies through its main policy indicator, the money supply. We did not use the prime rate as the economic policy variable, given that only Brazil and South Africa (among the 5 countries in the sample) have adopted the inflation targeting regime in 1999 and 2000 respectively. In this context, we use the monetary aggregates M1 and M2 as monetary policy variables. Future research should address this issue and see how the implementation of inflation targeting affects this result. The empirical specification used in this paper is standard in literature. However, there is a difference with respect to literature as the squared monetary policy variable is introduced, i.e., (Δ*M*/*GDP*)^2^, considering we've tested the monetary aggregates M1/GDP and M2/GDP. Thus, the hypothesis tested is that there is a nonlinear relationship between variations in bank lending and variations in the monetary aggregates (M1/GDP e M2/GDP). That said, the implications of this nonlinear relationship are analysed.

## Data and Methodological aspects

This paper employs bank-specific data from BankScope, a private database distributed by BVD-IBCA. We derive our data from four bank specializations—commercial, cooperative, savings and specialized government institutions (that act as a commercial bank). The balance sheet variables are converted into US dollars, which guarantees accounting uniformity among different countries. The use of unconsolidated financial statements avoids double-counting financial statements, since some banks may control others that are also present in Bankscope. We also employ macroeconomic data from the World Bank’s World Development Indicators (WDI). Data on the WDI are publicly available and can be downloaded at: http://databank.worldbank.org/data/download/WDI_excel.zip. After cleaning for missing, negative and outlier values of the main balance sheet variables we have in total 1059 banks, of which 76 are from Brazil, 117 from China, 62 from India, 789 from the Russian Federation, and 15 from South Africa. This final sample represents 68% of the pre-cleaning market share of assets in Brazil, 98% in China, 99% in India, 83% in Russia, and 99% in South Africa.

Table 1 shows the characteristics of the variables used in this article. Immediately after, we present the econometric specification that shows the variation of the bank loans as a function of the variation in our variable of interest ΔM and (ΔM)^2^, in addition to the usual control variables found in the literature.

**Table 1:** Definition of the variables.

Variables	Notes
Δ*Loans*_*it*_	Defined as ln(*loans*_*it*_) − ln(*loans*_*it*−1_). Loans is total loans net of provisions
$\Delta {\left(\frac{M1}{GDP}\right)}_{kt}$	Defined as $\text{ln}{\left(\frac{M1}{GDP}\right)}_{kt}-\text{ln}{\left(\frac{M1}{GDP}\right)}_{kt-1}$. Both M1 and GDP are nominal and in current local currencies*
$\Delta {\left(\frac{M2}{GDP}\right)}_{kt}$	Defined as $\text{ln}{\left(\frac{M2}{GDP}\right)}_{kt}-\text{ln}{\left(\frac{M2}{GDP}\right)}_{kt-1}$. Both M1 and GDP are nominal and in current local currencies*
Liquidity Ratio (*Liq*_*it*_)	Liquid assets to total assets ratio of bank *i* and year *t* (in %)
Market Share in Terms of Asses (*MS*_*it*_)	Market share in terms of assets
Equity Ratio_it_	Equity capital to total assets ratio of bank *i* and year *t* (in %)
Non Interest-Income (NII_it_)	Non-Interest income to total revenue ratio of bank *i* and year *t* (in %)
GDP HP Cycle	GDP gap of country *k* at year *t* calculated by applying a recursive HP filter to the natural log of GDP (Constant 2005 US$)*. We apply a HP filter using information available only up to the year in question
Consumer Price Index (CPI_it_)	Consumer price index of country *k* at year t*

Source: Prepared by authors.

We estimate the following equation:

$$\Delta Loans_{it}=\alpha_{0i}+\beta_{1}\Delta {\left(\frac{M}{GDP}\right)}_{kt}+\;\beta_{2}{\left(\Delta {\left(\frac{M}{GDP}\right)}_{kt}\right)}^{2}+\gamma_{1}Liq_{it}+\gamma_{2}MS_{it}+\gamma_{3}Equity\;Ratio_{it}+\;\gamma_{4}NII_{it}+\theta_{1}GDP\;Cycle_{kt}+\theta_{2}CPI_{kt}+\tau_{t}+\varepsilon_{it}$$ (1)

where we estimate five kinds of models: (a) a static fixed effects model with standard errors clustered at the country level; (b) a dynamic Pooled OLS model; (c) a dynamic fixed effects model; (d) an Arellano-Bond estimator, in which all the variables are considered endogenous and are thus instrumented by their own lags; and (e) and Arellano-Bond estimator where only the balance sheet variables are considered endogenous, while the country-specific variables are considered exogenous. In all specifications, we include standard errors clustered at the country level to allow for correlation among errors within country. We only employ instruments in differences for the endogenous variables in accordance with the instruments’ specification tests also presented in our main results tables. Note that M may refer to M1 or M2 aggregates.

## Empirical results

We present our results in Tables 2 and 3. In specifications [2] through [5], we add the lag of the dependent variable Δ*Loans*_*it*−1_ as an explanatory variable as well. In Tables 2 and 3 we also present a specification test.

**Table 2:** Regression of Bank’s Loan Growth on Monetary Expansion $\left(\Delta {\left(\frac{M1}{GDP}\right)}_{kt}\right)$ –BRICS.

	(1)	(2)	(3)	(4)	(5)
VARIABLES	Δ*Loans*_*it*_	Δ*Loans*_*it*_	Δ*Loans*_*it*_	Δ*Loans*_*it*_	Δ*Loans*_*it*_
Δ*Loans*_*it*−1_		0.148***	-0.110**	0.143***	0.104***
		(0.018)	(0.030)	(0.021)	(0.023)
$\Delta {\left(\frac{\text{M}1}{\text{GDP}}\right)}_{\text{kt}}$	0.529*	0.493**	0.485	0.496***	0.447***
	(0.246)	(0.155)	(0.252)	(0.064)	(0.082)
${\left(\Delta {\left(\frac{\text{M}1}{\text{GDP}}\right)}_{\text{kt}}\right)}^{2}$	-0.010**	-0.012	-0.013*	-0.012***	-0.030***
	(0.003)	(0.016)	(0.006)	(0.004)	(0.005)
*Liq*_*it*_	-0.450**	-0.040	-0.513**	-0.039	0.646***
	(0.130)	(0.062)	(0.179)	(0.026)	(0.109)
*MS*_*it*_	52.160	-37.220***	79.022	-36.202***	-84.394
	(152.879)	(7.411)	(163.129)	(6.313)	(74.885)
*Equity Ratio*_*it*_	-0.584***	-0.167***	-0.709***	-0.168***	0.001
	(0.096)	(0.030)	(0.120)	(0.038)	(0.168)
*NII*_*it*_	-0.030	-0.001	-0.052	-0.001	-0.100**
	(0.068)	(0.045)	(0.071)	(0.017)	(0.043)
GDP HP Cycle_it_	5.725***	4.998**	5.650***	5.035***	6.590***
	(1.035)	(1.179)	(1.004)	(0.305)	(0.397)
*CPI*_*it*_	1.244**	0.463	1.215**	0.470***	0.949***
	(0.433)	(0.338)	(0.398)	(0.137)	(0.260)
Constant	47.541***	30.683**	33.227***	31.887***	21.950***
	(4.866)	(8.351)	(3.789)	(1.828)	(3.452)
Observations	7,041	5,982	5,982	5,982	5,982
R-squared	0.284	0.235	0.301		
Number of Banks	1,059	1,051	1,051	1,051	1,051
Year Dummies	Yes	Yes	Yes	Yes	Yes
Method	Static FE	Dynamic Pooled OLS	Dynamic FE	Arellano Bond	Arellano Bond
Countries	BRICS	BRICS	BRICS	BRICS	BRICS
Endogenous Variables				Lag DEPVAR	Lag DEPVAR + Balance Sheet
Instruments Endogenous				Diff-Level	Diff-Level + Level
Exogenous Variables				All the others	All the others
Instruments Exogenous				Level	Level
AR(2) (P-value)				0.586	0.749
Hansen (P-value)				8.55e-10	0.156

Source: Prepared by authors.

Regarding to the significance level, the symbol *** stands for p<0.01, ** for p<0.05, and * for p<0.1.

**Table 3:** Regression of Bank’s Loan Growth on Monetary Expansion $\left(\Delta {\left(\frac{M2}{GDP}\right)}_{kt}\right)$ –BRICS.

	(1)	(2)	(3)	(4)	(5)
VARIABLES	Δ*Loans*_*it*_	Δ*Loans*_*it*_	Δ*Loans*_*it*_	Δ*Loans*_*it*_	Δ*Loans*_*it*_
Δ*Loans*_*it*−1_		0.157***	-0.102**	0.158***	0.124***
		(0.014)	(0.036)	(0.022)	(0.024)
$\Delta {\left(\frac{\text{M}2}{\text{GDP}}\right)}_{\text{kt}}$	0.948	0.714	0.962	0.716***	0.792***
	(0.655)	(0.411)	(0.604)	(0.107)	(0.132)
${\left(\Delta {\left(\frac{\text{M}2}{\text{GDP}}\right)}_{\text{kt}}\right)}^{2}$	-0.086**	-0.068	-0.076*	-0.068***	-0.093***
	(0.031)	(0.041)	(0.034)	(0.009)	(0.011)
*Liq*_*it*_	-0.456**	-0.038	-0.517**	-0.037	0.664***
	(0.122)	(0.063)	(0.172)	(0.026)	(0.110)
*MS*_*it*_	131.710	-41.743***	84.283	-41.272***	-44.713
	(146.153)	(4.251)	(160.862)	(6.130)	(49.142)
*Equity Ratio*_*it*_	-0.583***	-0.161**	-0.704***	-0.160***	-0.007
	(0.097)	(0.036)	(0.124)	(0.038)	(0.166)
*NII*_*it*_	-0.014	0.025	-0.036	0.025	-0.058
	(0.047)	(0.048)	(0.047)	(0.017)	(0.043)
GDP HP Cycle_it_	3.308**	2.832*	3.972**	2.826***	3.552***
	(1.056)	(1.321)	(1.216)	(0.429)	(0.477)
*CPI*_*it*_	1.268	0.546	1.487*	0.545***	1.065***
	(0.601)	(0.473)	(0.589)	(0.139)	(0.241)
Constant	29.784**	30.348**	51.961**	36.270***	24.801***
	(8.858)	(7.697)	(11.538)	(1.984)	(3.081)
Observations	7,041	5,982	5,982	5,982	5,982
R-squared	0.285	0.236	0.304		
Number of Banks	1,059	1,051	1,051	1,051	1,051
Year Dummies	Yes	Yes	Yes	Yes	Yes
Method	Static FE	Dynamic Pooled OLS	Dynamic FE	Arellano Bond	Arellano Bond
Countries	BRICS	BRICS	BRICS	BRICS	BRICS
Endogenous Variables				Lag DEPVAR	Lag DEPVAR + Balance Sheet
Instruments Endogenous				Diff-Level	Diff-Level + Level
Exogenous Variables				All the others	All the others
Instruments Exogenous				Level	Level
AR(2) (P-value)				0.625	0.815
Hansen (P-value)				0.000221	0.197

Source: Prepared by authors.

Regarding to the significance level, the symbol *** stands for p<0.01, ** for p<0.05, and * for p<0.1

In accordance with the banking related literature, we need to control for the possible correlation of the fixed effects with our variables of interest. We do so in specifications [1] (static) and [3] (dynamic). We also present a dynamic Pooled OLS estimation in specification [2], whose utility will become clear in a moment.

Table 2 presents the fixed-effects regression of loans (net of provisions) growth on several explanatory variables, among which is the growth of the ratio between M1 and the GDP. The subscript *t* refers to years, *i* to banks and *k* to countries. We drop the first and last percentile of the dependent variable for the whole sample to avoid outliers. Robust standard errors clustered by country in parentheses.

Table 2 shows that the negative coefficients of Δ(*M*1/*GDP*)_*kt*_ are all statistically significant at 10% level for columns [1] through [5], with the exception of column [3]. Meanwhile, the negative coefficients of [Δ(*M*1/*GDP*)]^2^_*kt*_ are statistically significant at 10% level for columns [1] through [5], except for column [3]. Hence, the empirical results show a nonlinear relationship between Δ*Loans* and Δ(*M*1/*GDP*) for the models described in columns [1], [4] and [5]. The estimated coefficients in columns [4] and [5] are statistically significant at 1% level. Along the same line, Table 3 also shows a nonlinear relationship between Δ*Loans* and Δ(*M*2/*GDP*) for the models described in columns [4] and [5] with an estimated coefficient at 1% level.

Table 3 presents the fixed-effects regression of loans (net of provisions) growth on several explanatory variables, among which is the growth of the ratio between M2 and the GDP. The subscript *t* refers to years, *i* to banks and *k* to countries. We drop the first and last percentile of the dependent variable for the whole sample to avoid outliers. Robust standard errors clustered by country in parentheses.

Note, however, that the problem of estimating dynamic specifications is that, in general, this variable is correlated with the fixed effects in the error term (leading to the “dynamic panel bias” [18]). Even if one controls for the fixed effects in the model, i.e., if one uses the within estimator, the endogeneity problem still persists: now the transformed lag of the dependent variable correlates with the transformed residual. The solution is to instrument the endogenous variables with their own lags.

The second lag of the dependent variable (Δ*Loans*_*t*−2_) would be a good instrument for Δ*Loans*_*t*−1_ = *Loans*_*t*−1_ − *loans*_*t*−2_, because it is correlated with it by construction and it is not correlated with the residual Δ*ε*_*t*_. The GMM estimator presents a good alternative to estimate this model since it allows overidentification (the use of more instruments than endogenous variables) and it directly addresses the case of non-spherical residuals.

In order to correct for this bias, we present specifications estimated using the method by [19]Arellano and Bond (1991), [20]Arellano and Bover (1995) and extended by [21]Blundell and Bond (1998) in columns [4] and [5]. [21]Blundell and Bond (1998) propose a system GMM estimator that combines the standard set of equations in first-differences with lagged levels as instruments, and an additional set of equation in levels with lagged first-differences as instruments to consistently estimate the model. Goodness of fit tests include: (i) the autocorrelation test: residuals in levels must not be auto-correlated, or residuals in differences must not present second order autocorrelation; (ii) good instruments: one must reject the Hansen J tests, whose null hypothesis states that the moments are close to zero. In addition, one should be careful with the number of instruments used [22](Roodman, 2009b). Too many instruments may lead to wrong inferences and unreliable specification tests.

Finally, according to [23]Roodman (2009a), one should expect that the coefficient of the lagged dependent variable, estimated using the Arellano and Bond (1991) estimator, be between the ones estimated by Pooled OLS (upward biased) and FE (downward biased) [1].

In columns [4] and [5] of both Tables, we use a forward orthogonal deviation transformation, as suggested by [19]Arellano and Bond (1991), so as to avoid magnifying the gaps in our panel (we have dropped the upper and lower 1% percentile). In addition, we show the p-values of the Hansen J test and second-order autocorrelation (AR(2)). One should not reject both tests in order to have goodness of fit. Note that while column [4] only considers as endogenous the lagged dependent variable, column [5] considers the balance-sheet variables as endogenous. In both cases, we only use the levels to instrument the exogenous variables (instrumented by themselves) and the balance-sheet variables (when they are considered endogenous). For the lagged case, we use both differences and levels. Both the AR(2) and the Hansen J tests point out that column [5] is well specified in Tables 2 and 3. Column [4], however, does not pass the Hansen J test in both Tables. Note, as well, that in both cases we have that the coefficients of the lagged dependent variable in column [4] and [5] are between the ones in column [2] (Pooled OLS) and column [3] (FE). However, we only suspect that the true population parameter for Δ*Loans*_*it*_ is positive, since the Pooled OLS coefficient is positive, while the FE coefficient is negative. Nevertheless, the former is higher than the latter, as expected by the theoretical bias of a positive coefficient for the lagged variable.

Of all the five specifications, column [5] is the one whose fitted values are more adherent to the real values. Results from this column show a positive and significant coefficient for the variation of the level of money supply and negative and significant coefficients for its squared term. This means that, in both, the effect of money supply growth on the growth of loans is non-linear and inverted U-shaped.

In the annex, we also include Tables 4 and 5 where we interact the linear and quadratic terms of real money balance growth for M1 and M2 with a crisis dummy (equal to one after 2007). Results in Tables 4 and 5 show that the inverted-U shaped relation between monetary expansion and bank loans is coming mostly from the crisis period (for M1, i.e. more liquid assets), while the same cannot be strongly said about M2 (which also includes near money assets). This shows that very strong QE policies during the crisis might have decreased risks, but they did not manage to make banks increase their loans more. The same can be said about very restrictive monetary policies, which is a more obvious conclusion.

**Table 4:** Regression of Bank’s Loan Growth on Monetary Expansion $\left(\Delta {\left(\frac{M1}{GDP}\right)}_{kt}\right)$ interacted with crisis dummy—BRICS.

	(1)	(2)	(3)	(4)	(5)
VARIABLES	Δ*Loans*_*it*_	Δ*Loans*_*it*_	Δ*Loans*_*it*_	Δ*Loans*_*it*_	Δ*Loans*_*it*_
Δ*Loans*_*it*−1_		0.147***	-0.115**	0.141***	0.103***
		(0.019)	(0.027)	(0.021)	(0.023)
$\Delta {\left(\frac{\text{M}1}{\text{GDP}}\right)}_{\text{kt}}$	0.954**	-0.037	1.637	-0.058	0.199
	(0.294)	(0.778)	(0.972)	(0.295)	(0.303)
${\left(\Delta {\left(\frac{\text{M}1}{\text{GDP}}\right)}_{\text{kt}}\right)}^{2}$	0.002	0.063	-0.044	0.066***	0.012
	(0.005)	(0.046)	(0.050)	(0.020)	(0.023)
$\Delta {\left(\frac{\text{M}1}{\text{GDP}}\right)}_{\text{kt}}\cdot Crisis_{t}$	-0.626**	0.346	-1.395	0.368	0.130
	(0.151)	(0.703)	(0.824)	(0.286)	(0.299)
${\left(\Delta {\left(\frac{\text{M}1}{\text{GDP}}\right)}_{\text{kt}}\right)}^{2}\cdot Crisis_{t}$	-0.022**	-0.082	0.026	-0.084***	-0.045**
	(0.008)	(0.040)	(0.050)	(0.021)	(0.023)
*Liq*_*it*_	-0.458**	-0.047	-0.529**	-0.045*	0.635***
	(0.122)	(0.056)	(0.162)	(0.027)	(0.110)
*MS*_*it*_	61.495	-36.518***	80.904	-35.526***	-80.376
	(144.393)	(7.296)	(158.064)	(6.494)	(71.217)
*Equity Ratio*_*it*_	-0.584***	-0.165***	-0.717***	-0.165***	0.004
	(0.095)	(0.028)	(0.111)	(0.038)	(0.167)
*NII*_*it*_	-0.016	0.010	-0.044	0.011	-0.089**
	(0.064)	(0.040)	(0.072)	(0.017)	(0.045)
GDP HP Cycle_it_	5.161***	4.524**	5.043***	4.559***	6.279***
	(0.887)	(1.003)	(0.960)	(0.306)	(0.419)
*CPI*_*it*_	0.824**	0.111	0.825**	0.119	0.713**
	(0.215)	(0.301)	(0.179)	(0.157)	(0.308)
Constant	44.638***	31.641**	35.413***	36.182***	24.640***
	(3.608)	(9.511)	(5.680)	(2.024)	(3.741)
Observations	7,041	5,982	5,982	5,982	5,982
R-squared	0.287	0.237	0.305		
Number of Banks	1,059	1,051	1,051	1,051	1,051
Year Dummies	Yes	Yes	Yes	Yes	Yes
Method	Static FE	Dynamic Pooled OLS	Dynamic FE	Arellano Bond	Arellano Bond
Countries	BRICS	BRICS	BRICS	BRICS	BRICS
Endogenous Variables				Lag DEPVAR	Lag DEPVAR + Balance Sheet
Instruments Endogenous				Diff-Level	Diff-Level + Level
Exogenous Variables				All the others	All the others
Instruments Exogenous				Level	Level
AR(2) (P-value)				0.613	0.773
Hansen (P-value)				5.02e-08	0.145

Source: Prepared by authors.

Regarding to table 4, the robust standard errors are given in parentheses and the symbol *** stands for p<0.01, ** for p<0.05, and * for p<0.1.

**Table 5:** Regression of Bank’s Loan Growth on Monetary Expansion $\left(\Delta {\left(\frac{M2}{GDP}\right)}_{kt}\right)$ interacted with crisis dummy—BRICS.

	(1)	(2)	(3)	(4)	(5)
VARIABLES	Δ*Loans*_*it*_	Δ*Loans*_*it*_	Δ*Loans*_*it*_	Δ*Loans*_*it*_	Δ*Loans*_*it*_
Δ*Loans*_*it*−1_		0.154***	-0.105**	0.158***	0.110***
		(0.012)	(0.033)	(0.027)	(0.026)
$\Delta {\left(\frac{\text{M}2}{\text{GDP}}\right)}_{\text{kt}}$	1.668	1.114	2.891*	-0.350	16.089
	(0.881)	(1.105)	(1.047)	(17.624)	(10.239)
${\left(\Delta {\left(\frac{\text{M}2}{\text{GDP}}\right)}_{\text{kt}}\right)}^{2}$	-0.068	0.024	-0.120	0.071	-1.074
	(0.038)	(0.081)	(0.071)	(1.120)	(0.657)
$\Delta {\left(\frac{\text{M}2}{\text{GDP}}\right)}_{\text{kt}}\cdot Crisis_{t}$	-0.915	-0.649	-2.184	0.952	-15.724
	(0.817)	(1.467)	(1.240)	(18.129)	(10.548)
${\left(\Delta {\left(\frac{\text{M}2}{\text{GDP}}\right)}_{\text{kt}}\right)}^{2}\cdot Crisis_{t}$	-0.013	-0.091	0.051	-0.140	0.997
	(0.019)	(0.065)	(0.057)	(1.139)	(0.668)
*Liq*_*it*_	-0.462**	-0.050	-0.526**	-0.043	0.649***
	(0.114)	(0.051)	(0.162)	(0.032)	(0.116)
*MS*_*it*_	134.837	-42.349***	82.743	-40.119*	-80.624
	(141.979)	(3.722)	(157.680)	(21.028)	(64.370)
*Equity Ratio*_*it*_	-0.587***	-0.164***	-0.716***	-0.161***	-0.018
	(0.092)	(0.030)	(0.112)	(0.041)	(0.166)
*NII*_*it*_	-0.005	0.030	-0.029	0.029	-0.068
	(0.041)	(0.043)	(0.042)	(0.018)	(0.046)
GDP HP Cycle_it_	2.850*	2.040	3.377*	2.372**	3.202***
	(1.182)	(1.500)	(1.331)	(0.955)	(0.917)
*CPI*_*it*_	0.889	0.127	1.044	0.304	0.938*
	(0.532)	(0.444)	(0.558)	(0.466)	(0.515)
Constant	26.099**	29.738**	60.832***	38.836***	27.302***
	(8.885)	(9.199)	(11.425)	(5.484)	(5.967)
Observations	7,041	5,982	5,982	5,982	5,982
R-squared	0.286	0.240	0.307		
Number of Banks	1,059	1,051	1,051	1,051	1,051
Year Dummies	Yes	Yes	Yes	Yes	Yes
Method	Static FE	Dynamic Pooled OLS	Dynamic FE	Arellano Bond	Arellano Bond
Countries	BRICS	BRICS	BRICS	BRICS	BRICS
Endogenous Variables				Lag DEPVAR	Lag DEPVAR + Balance Sheet
Instruments Endogenous				Diff-Level	Diff-Level + Level
Exogenous Variables				All the others	All the others
Instruments Exogenous				Level	Level
AR(2) (P-value)				0.577	0.882
Hansen (P-value)				6.26e-05	0.313

Source: Prepared by authors.

Regarding to table 5, the robust standard errors are given in parentheses and the symbol *** stands for p<0.01, ** for p<0.05, and * for p<0.1.

## Conclusions

This article examines the bank lending channel, which considers how monetary authority actions affect the variation of loans. We focus on the BRICS (Brazil, Russia, India, China and South Africa) totalizing 1254 banks from 5 countries in the period 2000–2012 (totalizing 13 years). The central bank influences the variation of loan supplies through its main indicator of policy rate. The empirical specification used in this paper is standard in literature. However, there is a difference with respect to literature as the squared monetary policy variable is introduced, i.e., [(Δ(*M*/*GDP*)]^2^.

Results show a positive and significant coefficient for the variation of money supply and negative and significant coefficients for its squared term. In short, the results show empirical evidence that there is a nonlinear relationship between variations in bank loans and interest rate variations. This means that, in both, the effect of money supply growth on the growth of loans is non-linear and inverted U-shaped. Therefore, the marginal effect of money growth on loans is positive for values lower than the maximum point and negative for values higher than this same point.

In this context, our results show empirical evidence that in the case of the BRICS, in the period from 2000 to 2012, expansionary monetary policies do not increase the propensity of economic agents to systematically take greater risks on the market. For some reason, either via institutional bank regulation, or due to the market’s perception of greater credit risks, it can be said that on average, from a certain level of money stock, increased money supply does not induce the increase of negotiated credit.
